# Fibroblast activation protein-α-expressing fibroblasts promote the progression of pancreatic ductal adenocarcinoma

**DOI:** 10.1186/s12876-015-0340-0

**Published:** 2015-09-02

**Authors:** Tomoya Kawase, Yumiko Yasui, Sohji Nishina, Yuichi Hara, Izumi Yanatori, Yasuyuki Tomiyama, Yoshihiro Nakashima, Koji Yoshida, Fumio Kishi, Masafumi Nakamura, Keisuke Hino

**Affiliations:** 1 Department of Hepatology and Pancreatology, Kawasaki Medical School, 577 Matsushima, Kurashiki, Okayama 701-0192 Japan; 2 Department of Molecular Genetics, Kawasaki Medical School, Kurashiki, Japan; 3 Department of Digestive Surgery, Kawasaki Medical School, Kurashiki, Japan; 4 Department of Surgery and Oncology, Graduate School of Medical Sciences Kyusyu University, Fukuoka, Japan

## Abstract

**Background:**

Pancreatic ductal adenocarcinoma (PDAC) is characterized by an extensive desmoplastic stromal response. Fibroblast activation protein-α (FAP) is best known for its presence in stromal cancer-associated fibroblasts (CAFs). Our aim was to assess whether FAP expression was associated with the prognosis of patients with PDAC and to investigate how FAP expressing CAFs contribute to the progression of PDAC.

**Methods:**

FAP expression was immunohistochemically assessed in 48 PDAC specimens. We also generated a fibroblastic cell line stably expressing FAP, and examined the effect of FAP-expressing fibroblasts on invasiveness and the cell cycle in MiaPaCa-2 cells (a pancreatic cancer cell line).

**Results:**

Stromal FAP expression was detected in 98 % (47/48) of the specimens of PDAC, with the intensity being weak in 16, moderate in 19, and strong in 12 specimens, but was not detected in the 3 control noncancerous pancreatic specimens. Patients with moderate or strong FAP expression had significantly lower cumulative survival rates than those with negative or weak FAP expression (mean survival time; 352 vs. 497 days, *P* = 0.006). Multivariate analysis identified moderate to strong expression of FAP as one of the factors associated with the prognosis in patients with PDAC. The intensity of stromal FAP expression was also positively correlated to the histological differentiation of PDAC (P < 0.05). FAP-expressing fibroblasts promoted the invasiveness of MiaPaCa-2 cells more intensively than fibroblasts not expressing FAP. Coculture with FAP-expressing fibroblasts significantly activated cell cycle shift in MiaPaCa-2 cells compared to coculture with fibroblasts not expressing FAP. Furthermore, coculture with FAP expressing fibroblasts inactivated retinoblastoma (Rb) protein, an inhibitor of cell cycle progression, in MiaPaCa-2 cells by promoting phosphorylation of Rb.

**Conclusions:**

The present *in vitro* results and the association of FAP expression with clinical outcomes provide us with a better understanding of the effect of FAP-expressing CAFs on the progression of PDAC.

## Background

Pancreatic ductal adenocarcinoma (PDAC) is characterized by an aggressive course, early metastasis, and a limited response to chemotherapy and radiotherapy, resulting in the overall 5-year survival rate of less than 5 % [1–4]. In many solid tumors, the stroma is increasingly recognized to be important in promoting tumor proliferation, invasion, metastasis, and chemoresistance [5]. PDAC is also characterized by an extensive desmoplastic stromal response. Cancer-associated fibroblasts (CAFs) are currently recognized to be fibroblasts that acquire an activated phenotype within the tumor stroma [6]. Mounting evidence suggests that CAFs actively communicate with and stimulate tumor cells, thereby contributing tumor development and progression [6–8].

Fibroblast activation protein-α (FAP) is a 95-kDa cell surface glycoprotein belonging to the serine protease family that cleaves the peptide bound between proline and other amino acids, and this activity modifies various bioactive molecules [9]. Homodimerization to a 170-kDa form is necessary for the dipeptidyl peptidase and gelatinase activities [10]. FAP is best known for its presence in stromal CAFs, found in over 90 % of epithelial tumors [11, 12], even though it is also expressed in reactive fibroblasts during embryonic development, wound healing, chronic inflammation and in cancer cells [13–16]. Recent studies have demonstrated that FAP expressed in stromal CAFs has a critical role in the clinical outcomes of patients with PDAC [12, 16]. In addition, some biological properties of FAP such as matrix production supportive for cell motility, immune suppression, and angiogenesis during the extensive desmoplastic response associated with this cancer have been demonstrated [17–20]. However, it remains to be elucidated how FAP-expressing CAFs contribute to the disease progression of PDAC. The aim of this study was to assess the relation of FAP expression in CAFs to overall survival in patients with PDAC and to investigate the mechanisms by which FAP activates tumor progression in PDAC.

## Methods

### Operated pancreatic specimens

PDAC specimens were obtained from 48 patients who had undergone surgical resection for PDAC at Kawasaki Medical School Hospital from 2006 to 2012. The study protocol conformed to the 1975 Helsinki declaration, and was approved by the Research Ethics Committee of Kawasaki Medical School (Admission No: 894-1). The need for informed consent was waived by the Research Ethics Committee, because the study was retrospective and some patients had already been dead. Three resected noncancerous pancreatic specimens were used as controls (one chronic pancreatitis tissue and two normal pancreatic tissues that were resected due to bile duct cancer and duodenal papillary cancer). The clinical characteristics of the patients were as follows: age, 71.5 ± 1.3; gender, 28 males (58 %); clinical stage of PDAC based on the TNM classification of the Union for International Cancer Control, 7 in stage I, 16 in stage II, 16 in stage III, and 9 in stage IV. Alcohol intake was defined as 37.5 g/day or more on the basis of alcohol intake and pancreatic cancer risk deduced from a meta-analysis of the dose-risk relation [21]. None of the patients underwent preoperative chemotherapy, but 37 patients underwent postoperative chemotherapy. All patients were followed up after operation and survival time was defined as the interval between the diagnosis of PDAC and death or the last visit to the outpatient clinic up to March 31, in 2013.

### Immunohistochemistry

The FAP-positive cells in paraffin-embedded specimens were identified by immunohistochemical staining using a rabbit anti-human Fibroblast activation protein, alpha antibody (ab53066) (Abcam, Cambridge, MA), as described previously [12].

### Cell culture

MiaPaCa-2 and BxPC-3 pancreatic tumor cells and NIH-3 T3 fibroblasts were obtained from DS Pharma Biomedical (Osaka, Japan). MiaPaCa-2 and NIH-3 T3 were cultured in DMEM, and BxPC-3 in RPMI 1640, at 37 °C in a humidified atmosphere with 5 % CO_2_. Both media contained 10 % fetal bovine serum (FBS).

### Cloning of human fap gene and cell transfection

The human *fap* (*hfap*) gene was amplified by polymerase chain reaction (PCR) using BxPC-3 genome as a template and specific *hfap* primers that were deduced from the NCBI reference sequence (NM_004460.3): fw_5’- AGATCTATGAAGACTTGGGTAAAAATCGTA-3’ and rev_5’-AGATCTTTAGTCTGGTCTACAAAGAGAAAACACTG-3’ (the incorporated *Bgl*II sites are underlined). The resulting PCR products were cloned into the vector pUC13, purified, sequenced, digested at the *Bgl*II site, and subcloned in the pIRESneo3 (Clontech, Mountain View, CA) vector. NIH-3 T3 cells were transfected with the pIRESneo3 vector containing the hFAP cDNA or pIRESneo3 vector using FuGENE® 6 Transfection Reagent (Roche Applied Science, Mannheim, Germany) according to the manufacturer’s instructions, followed by selection with 400 μg/mL G418.

### Invasion assay

Migration of MiaPaCa-2 cells was examined by the two-chamber assay using a CultureCoat® 24 Well High BME Cell Invasion Assay (Trevigen, Gaithersburg, MD), as described previously [22]. Briefly, NIH-3 T3 fibroblasts with/without FAP expression (5×10^4^ cells/well) were seeded into 24-well plates. MiaPaCa-2 pancreatic cancer cells (5×10^4^ cells/well) were seeded into culture inserts (8 μm pores) and placed on 24-well plates containing NIH-3 T3 fibroblasts. Both types of cells were cultured in DMEM supplemented with 10 % FBS. After 24-h incubation, the cell suspension in the upper chamber was aspirated and the upper surface of the polycarbonate membrane was carefully cleaned with cotton plugs. Cells that migrated through the membrane were put on a glass slide, stained, and counted in 4 randomly selected fields/well in 4 wells at x100 magnification. The polycarbonate membrane is coated with a thick basement membrane that is intended for use highly invasive cell lines.

### *In vitro* coculture system

Using the same culture plates and culture inserts as those used in invasion assay, MiaPaCa-2 cells (1×10^5^ cells/well) were seeded in 6-well culture plates (Becton Dickinson, Franklin Lakes, NJ). NIH-3 T3 fibroblasts with/without FAP expression (1×10^5^ cells/well) were seeded into the culture inserts (8 μm pores) and placed on 6-well culture plates containing MiaPaCa-2 cells. Both cell types were cultured in DMEM supplemented with 10 % FBS.

### Cell cycle analysis

After 24-h *in vitro* coculture, MiaPaCa-2 cells were fixed with formalin, incubated for 30 min with DNA Dye (Hoechst 33342), and rinsed with PBS. Plates containing the cells were scanned with the ImageXpress Micro Screening System (Molecular Devices, Tokyo, Japan). Classification of cell phases was based on the fluorescence intensity of DNA dye. For cell number and cell cycle analyses, the intensity of the integrated DNA dye was assessed using the cell-cycle application module (MetaXpress, Molecular Devices), as described previously [23].

### Western blotting

Cell lysates were separated by sodium dodecyl sulfate-polyacrylamide gel electrophoresis. The proteins were transferred to polyvinylidene difluoride membranes (Pall Corporation, New York, NY), blocked overnight at 4 °C with 5 % skim milk and 0.1 % Tween 20 in Tris-buffered saline, and subsequently incubated for 2-h at room temperature with a mouse monoclonal antibody raised against a partial recombinant FAP (Abnova, Taipei, Taiwan), a rabbit anti-human Phospho-Rb (Ser807/811) antibody (Cell Signaling Technology, Danvers, MA) and mouse monoclonal anti-α-tubulin antibody (Sigma-Aldrich, St. Louis, MO).

### Statistical analysis

Continuous variables were expressed as mean ± SD. Comparison between groups were performed using the *χ*^2^ test for categorical variables. Cumulative survival was calculated using the Kaplan-Meier method and the differences among the groups were analyzed with the log-rank test. Univariate and multivariate analyses of predictors of survival were assessed using the Cox proportional hazards model. A *P* value of less than 0.05 was considered to be significant. All analyses described above were performed using SPSS software (version 11, SPSS Inc., Chicago, IL).

## Results

### Stromal FAP expression in resected PDAC

**Fig. 1:**
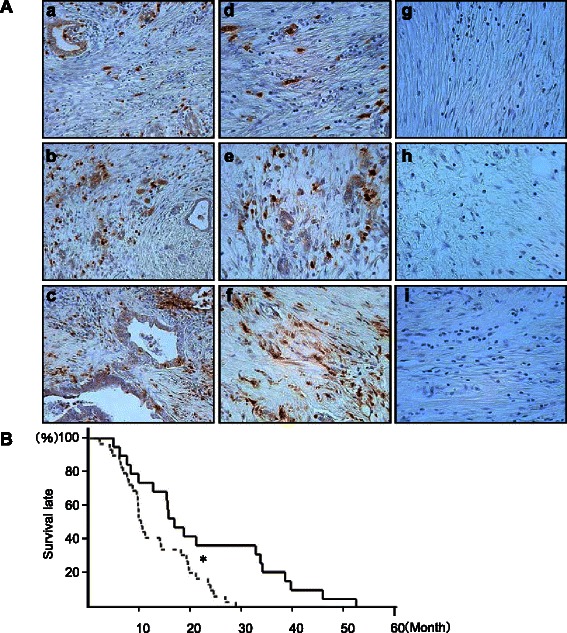
Stromal fibroblast activation protein-α (FAP) expression in resected pancreatic ductal adenocarcinoma (PDAC), and relationship between intensity of FAP expression and cumulative survival in patients with PDAC. **A** The expression of FAP was found predominantly in stromal cells and slightly in cancer cells (a-c, x200 field magnification). Stromal fibroblast FAP expression was graded by the number of positive cells per 1000 stromal fibroblasts for three randomly selected views (weak < 350 [d], 350 ≤ moderate < 650 [e], and 650 ≤ strong [f]), but negative in chronic pancreatitis (g) and noncancerous pancreatic tissues that were resected due to bile duct cancer (h) and duodenal papillary cancer (i) (d-i, x400 field magnification). **B** Cumulative survival curves for PDAC patients with negative or weak FAP expression and those with moderate or strong FAP expression. The solid line and broken lines indicate patients with negative or weak FAP expression and those with moderate or strong FAP expression, respectively. Log-rank test *P* = 0.006

The expression of FAP was found predominantly in stromal cells and slightly in cancer cells in resected PDAC tissues. In the present study we focused on stromal FAP expression in terms of the effect of stromal FAP expression on pancreatic cancer cells, and regarded FAP expressing stromal fibroblasts as CAFs. Stromal FAP expression was exclusively found in fibroblasts and graded by the number of positive cells per 1000 stromal fibroblasts for three randomly selected views (negative, weak < 350, 350 ≤ moderate < 650, and 650 ≤ strong), as shown in Fig. 1A. Stromal FAP expression was detected in 98 % (47/48) of the specimens of PDAC, with the intensity being weak in 16, moderate in 19, and strong in 12 specimens, but was not detected in the 3 control specimens. Patients with moderate or strong FAP expression had significantly lower cumulative survival rates than those with negative or weak FAP expression (mean survival time; 352 vs. 497 days, *P* = 0.006) (Fig. 1B). Multivariate analysis identified moderate to strong stromal FAP expression, distant metastasis and alcohol intake as significant factors associated with overall survival in patients with PDAC (Table 1). The intensity of stromal FAP expression was also significantly correlated to the histological differentiation of PDAC (Table 2).

**Table 1:** Factors associated with overall survival in operated patients with PDAC

	Univariate analysis			Multivariate analysis		
Factor	Hazard ratio	95 % CI	*P* value	Hazard ratio	95 % CI	*P* value
Age (≥65)			0.631			
Gender			0.384			
Smoking			0.206			
Alcohol	2.082	1.103-3.930	0.024	1.941	1.024-3.678	0.042
DM			0.966			
Chemotherapy^a^			0.219			
Tumor factor						
T3 or T4^b^			0.513			
N1^b^			0.115			
M1^b^	2.863	1.072-7.646	0.036	2.972	1.097-8.055	0.032
Stage III or IV^c^			0.271			
Histological factor						
ly^1^			0.659			
v^2^			0.195			
n^3^			0.270			
FAP moderate/strong	2.540	1.273-5.068	0.008	2.534	1.267-5.068	0.009

^a^postoperative, ^b^TNM classification of Union for International Cancer Control (UICC), ^c^clinical stage of PDAC by UICC, ^1^lymph duct invasion, ^2^vascular invasion, ^3^nerve invasion

**Table 2:** Relationship between stromal FAP expression and clinicopathological factors

Characteristics	FAP negative/weak	FAP moderate/strong	*P* value
Age			0.911
≥65	16	26	
<65	3	3	
T category^a^			0.479
T1/T2	5	4	
T3/T4	14	25	
N category^a^			0.444
N0	10	12	
N1	9	17	
M category^a^			0.317
M0	17	22	
M1	2	7	
UICC^b^ stage			0.406
I/II	11	13	
III/IV	8	16	
Histological differentiation			<0.05
Well	6	2	
Moderate/Poor	13	27	

^a^TNM classification of the UICC, ^b^UICC, Union for International Cancer Control

### Effect of FAP-expressing fibroblasts on invasiveness of MiaPaCa-2 cells

**Fig. 2:**
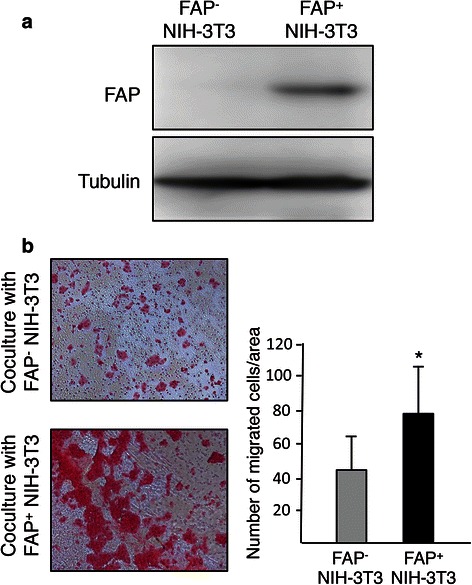
Effect of fibroblast activation protein-α (FAP) expressing NIH-3 T3 cells on invasiveness of MiaPaCa-2 cells. **a** Western blot analysis shows stable expression of FAP in NIH-3 T3 cells. **b** MiaPaCa-2 cells that were cocultured with NIH-3 T3 cells with/without FAP expression and then migrated through the polycarbonate membrane were stained with H&E, and counted in 4 randomly selected fields/well in 4 wells at x100 field magnification. The invasion assay was performed 13 times. *, *P* < 0.01

To investigate the mechanism by which stromal FAP expression promoted the progression of PDAC, we established NIH-3 T3 cells that stably expressed human FAP (Fig. 2a). Increased invasiveness is one of the features that support the rapid progression of PDAC. We then examined effect of FAP-expressing fibroblasts on the invasiveness of MiaPaCa-2 cells using an invasion assay in a coculture system. The number of MiaPaCa-2 cells that migrated through the polycarbonate membrane was significantly greater in coculture with FAP-expressing NIH-3 T3 cells than in coculture with NIH-3 T3 cells not expressing FAP (Fig. 2b). There was no direct contact between cells in culture plates and cells in culture inserts. Thus, indirect coculture with FAP-expressing NIH-3 T3 cells promoted the invasiveness of MiaPaCa-2 cells more intensively than that with NIH-3 T3 cells not expressing FAP. These results suggested that FAP-expressing fibroblasts promoted the invasiveness of pancreatic cancer cells and were consistent with the epithelial-mesenchymal transition promoted by pancreatic stellate cells [21] and the increased invasive velocity of FAP-overexpressing fibroblasts [17] in previous reports.

### Effect of FAP-expressing fibroblasts on cell cycle of MiaPaCa-2 cells

**Fig. 3:**
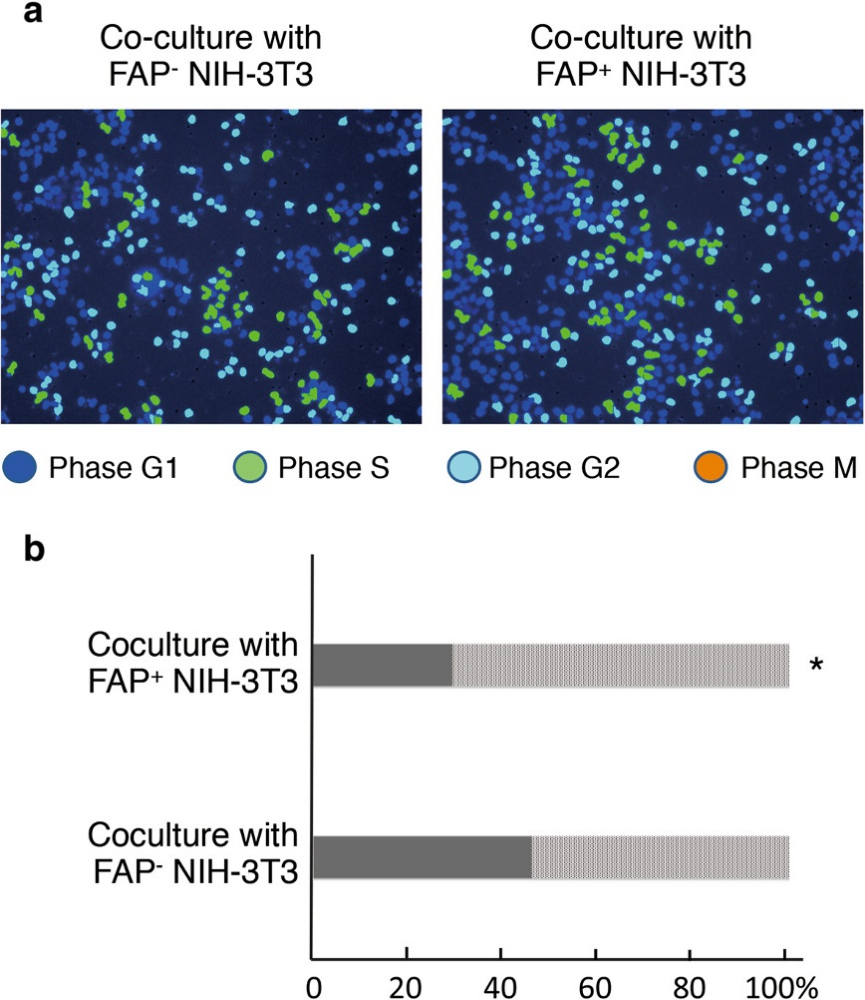
Cell cycle analysis of MiaPaCa-2 cells after coculture with NIH-3 T3 cells with/without FAP expression. **a** MiaPaCa-2 cells that were cocultured with NIH-3 T3 cells with/without FAP expression were examined for cell cycle distribution by using the integrated fluorescence intensity, which reflects cellular DNA content with the ImageXpress Micro Screening System. **b** Cell cycle distribution in MiaPaCa-2 cells that were cocultured with NIH 3 T3 cells with/without FAP. The experiments were repeated four times. Dark gray bars and bright gray bars indicate the G0/G1 phase fraction and S/G2/M phase fraction, respectively. *, *P* < 0.05

The significant correlation between stromal FAP expression and histological differentiation of PDAC prompted us to investigate whether stromal FAP expression affected the cell cycle of pancreatic cancer cells. MiaPaCa-2 cells, which were cocultured with NIH-3 T3 cells with/without FAP expression, were examined for cell cycle distribution by using the integrated fluorescence intensity which reflected the cellular DNA content (Fig. 3a). Coculture with FAP-expressing NIH-3 T3 cells significantly decreased the fraction of cells in the G0/G1 phase in MiaPaCa-2 cells compared with coculture with NIH-3 T3 cells not expressing FAP (Fig. 3b). These results suggested that coculture with FAP expressing NIH-3 T3 cells activated switching from G0/G1 to S/G2/M in MiaPaCa-2 cells.

### Inactivation of retinoblastoma (Rb) by FAP-expressing fibroblasts

**Fig. 4:**
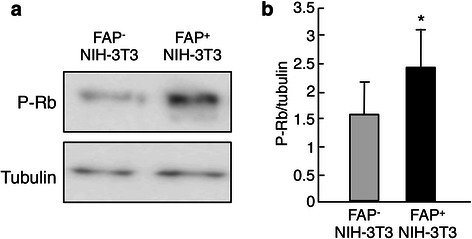
Phosphorylation of Rb protein in MiaPaCa-2 cells that were cocultured with NIH-3 T3 cells with/without FAP. **a** Immunoblots for phosphorylated Rb (P-Rb) using MiaPaCa-2 cell lysates that were cocultured with NIH 3 T3 cells with/without FAP. **b** The P-Rb expression level was normalized to that of tubulin. The experiments were repeated three times. The black and gray bars indicate MiaPaCa-2 cells after coculture with FAP-expressing NIH-3 T3 cells and MiaPaCa-2 cells after coculture with NIH-3 T3 cells without FAP expression, respectively. *, *P* < 0.05

Rb protein is an inhibitor of cell cycle progression, that is, Rb arrests cells in G1 phase. Rb is phosphorylated and dephosphorylated during the cell cycle; the hyperphosphorylated form predominates in proliferating cells, whereas the hypophosphorylated form is generally more abundant in quiescent or differentiating cells. Rb binds to a gene regulatory protein called E2F and blocks the transcription of S-phase genes. Phosphorylated Rb reduces its affinity for E2F, and then dissociates, allowing E2F to activate S-phase gene expression [24]. Therefore, we next examined the phosphorylation of Rb to clarify the mechanisms underlying switching from G0/G1 to S/G2/M in MiaPaCa-2 cells cocultured with FAP-expressing NIH-3 T3 cells. MiaPaCa-2 cells cocultured with FAP-expressing NIH-3 T3 cells showed significantly increased phosphorylation of Rb compared to those cocultured with NIH-3 T3 cells not expressing FAP (Fig. 4). These results suggested that coculture with FAP-expressing NIH-3 T3 cells promoted phosphorylation of Rb and subsequently activated the cell cycle shift from G0/G1 to S/G2/M in MiaPaCa-2 cells.

## Discussion

In the present study moderate to strong stromal FAP expression, distant metastasis and alcohol intake was identified as significant factors associated with overall survival in patients with PDAC. These results are in part untypical in terms of no correlation for post-operative chemotherapy, tumor size, lymph node involvement, but for alcohol intake. Heavy alcohol intake (≥37.5 g/day) and relatively low proportion (18.7 %) of T1/T2 stage tumors in our study population might be related to these untypical results, even though the precise explanation remains elusive. In addition, it needs to be careful for interpretation of the results that the intensity of stromal FAP expression was significantly correlated to the histological differentiation of PDAC, because the majority of cases in both FAP expression and histological differentiation of PDAC are not well differentiated. Therefore, while FAP expression may play a role in determining histological differentiation, it does not seem to be the dominant factor for determining histological differentiation. It has been reported that FAP is expressed exclusively in stromal fibroblasts of epithelial cancers [12, 25–27], though some reports have suggested that FAP is expressed in both the stromal and epithelial compartments of cancers [16, 28–30]. Although this issue is still controversial, we successfully amplified the hFAP cDNA by PCR using BxPC-3 (a pancreatic cancer cell line) mRNA as a template and specific *hfap* primers, and established NIH-3 T3 cells that stably expressed human FAP. The reason we used BxPC-3 mRNA as the template for amplifying *hfap* was the immunoblotting detection of FAP in BxPC-3 cells in a previous study [16]. TGF-β is a powerful factor for inducing FAP expression in NIH-3 T3 fibroblasts [31] and, therefore, induction of FAP expression by TGF-β may be more suitable in terms of mimicking the environment of the stromal response to cancer in human PDAC tissue. However, we chose to establish fibroblasts stably expressing FAP to avoid fluctuation in the expression level of FAP under our experimental conditions. The use of cells of different species (NIH-3 T3 cells generated from embryonic mouse fibroblasts and MiaPaCa-2 cells from human pancreatic cancer) may not be a confounding factor, because human FAP, not mouse FAP, was expressed in NIH-3 T3 cells.

The present findings that stromal FAP expression in resected PDAC specimens was negatively correlated with patients’ overall survival was consistent with the association of higher FAP expression with worse clinical outcomes in patients with PDAC in previous studies [12, 16]. We identified at least two mechanisms that accounted for these results. One was the effect of FAP on the invasiveness of pancreatic cancer cells. FAP-expressing NIH-3 T3 cells promoted the invasiveness of MiaPaCa-2 cells more intensively than NIH-3 T3 cells not expressing FAP without direct contact between cells. Antiplasmin-cleaving enzyme (APCE) has been identified as a soluble form of FAP, resulting from cleavage of the Cys23-Ile24 bond in the transmembrane or extracellar domain [32]. Therefore, it is reasoned that FAP-expressing fibroblasts promoted the invasiveness of MiaPaCa-2 cells even in the absence of direct contact of these two cells, even though we could not measured the APCE level in culture medium. The extracellular matrix is composed of glycoproteins, collagen, and proteases that modify structural proteins and regulate clotting factors that can facilitate pancreatic cancer cell invasion [33]. FAP-overexpressing fibroblasts have been shown to produce an extracellular matrix that enhances the invasive velocity and directionality of pancreatic cancer cells [17]. In addition, pancreatic stellate cells obtained from the resected pancreatic tissues of patients with pancreatic cancer have been shown to increase the migration of pancreatic cancer cells in coculture system [22]. These results are well consistent with the increased invasiveness of MiaPaCa-2 cells in coculture with FAP-expressing NIH-3 T3 cells in this study.

The other mechanism is the activation of cell cycle progression by FAP-expressing fibroblasts. To our knowledge, this is the first report to demonstrate that FAP-expressing fibroblasts promote phosphorylation of Rb and subsequently activate cell cycle progression in pancreatic cancer cells. When cells are stimulated to divide by extracellular signals, active G_1_-cyclin-dependent protein kinase (Cdk) accumulates and phosphorylates Rb, reducing its affinity for E2F. Therefore, further studies including assessment of the effect of FAP on G_1_-Cdk expression/activity are required to clarify how FAP-expressing fibroblasts activate phosphorylation of Rb protein in pancreatic cancer cells. The activated cell cycle shift from G0/G1 to S/G2/M in pancreatic cancer cells cocultured with FAP-expressing fibroblasts may be one of the important mechanisms underlying the significant correlation between the intensity of stromal FAP expression and the histological differentiation of cancer cells in patients with PDAC (Table 2). On the other hand, FAP expression has been reported in mesenchymal stem cells [34, 35]. Mesenchymal stem cells within tumor stroma have been shown to promote tumor growth and metastatic capacity of cancer cells [36, 37]. These results seem to provide an interesting link between FAP expression and the differentiation state of PDAC. Albeit with data from only one cell line, our *in vitro* results and the association of FAP expression with clinical outcomes provides us with a better understanding of the influence of FAP-expressing CAFs on epithelial tumor cell behavior and targeted therapeutics aimed at disrupting specific tumor-stromal interactions. In addition to the activation of invasiveness and/or cell cycle progression, FAP-expressing fibroblasts have a role in immune suppression [18] and angiogenesis during the extensive desmoplastic response associated with this cancer [19, 20]. Thus, FAP-expressing fibroblasts are critical for remodeling a permissive stromal environment that supports pancreatic cancer progression.

## Conclusion

Moderate to strong expression of FAP was one of the factors associated with the prognosis in patients with PDAC and the intensity of stromal FAP expression was positively correlated to the histological differentiation of PDAC. As the mechanisms underlying these clinical results, FAP-expressing fibroblasts promoted the invasiveness and activated cell cycle of a pancreatic cancer cell line *in vitro*.

## Abbreviations

PDAC: Pancreatic ductal adenocarcinoma
FAP: Fibroblast activation protein-α
CAFs: Cancer-associated fibroblasts
Rb: Retinoblastoma
Cdk: G_1_-cyclin-dependent protein kinase
APCE: Antiplasmin-cleaving enzyme
